# A journal club for peer mentorship: helping to navigate the transition to independent practice

**DOI:** 10.1007/s40037-016-0292-2

**Published:** 2016-09-08

**Authors:** Thomas E. MacMillan, Shail Rawal, Peter Cram, Jessica Liu

**Affiliations:** 1 Division of General Internal Medicine, University Health Network, Toronto, ON Canada; 2 Division of General Internal Medicine, Department of Medicine, University of Toronto, Toronto, ON Canada; 3 Sinai Health System, Toronto, ON Canada

**Keywords:** Mentorship, Medical education, Transition to practice, Residency, Journal club, Continuing medical education

## Abstract

The transition from residency to independent practice presents unique challenges for physicians. New attending physicians often have unmet learning needs in non-clinical domains. An attending physician is an independent medical practitioner, sometimes referred to as a staff physician or consultant. Peer mentorship has been explored as an alternative to traditional mentorship to meet the learning needs of new attendings. In this article, the authors describe how a journal club for general internal medicine fellowship graduates helped ease the transition by facilitating peer mentorship. Journal club members were asked to bring two things to each meeting: a practice-changing journal article, and a ‘transition to practice’ discussion topic such as a diagnostic dilemma, billing question, or a teaching challenge. Discussions fell into three broad categories that the authors have termed: trading war stories, measuring up, and navigating uncharted waters. It is likely that physicians have a strong need for peer mentorship in the first few years after the transition from residency, and a journal club or similar discussion group may be one way to fulfil this.

The transition from trainee to practising physician is an exciting and daunting time. Whether entering academic or community practice, the change can be abrupt and isolating. In a teaching hospital, attending physicians typically lead a team of medical students and residents on a clinical teaching unit. An attending physician is an independent medical practitioner, sometimes referred to as a staff physician or consultant. At the completion of training, new attending physicians are more confident with their clinical knowledge than with non-clinical domains such as communication, leadership, and teamwork [1, 2]. New attendings experience stress when managing financial issues, conflict resolution with colleagues, performance targets, and work-life balance [3]. They often feel unprepared for negotiation, business planning, medicolegal issues, and research [4]. New attendings may be given supervisory and teaching roles despite insufficient training [5, 6].

Access to mentorship may ease the transition for new attendings [5]. In a traditional mentorship relationship, a senior colleague (the mentor) may take on the role of teacher, advisor, coach, or confidante to a junior colleague (the mentee) [7, 8]. Recently, peer mentorship has been explored as an alternative to traditional one-way mentorship [1, 2]. In peer mentorship, individuals with a similar level of experience mentor each other [8, 9]. Peer mentorship can occur between two people or in a group [8]. Group peer mentorship may be informal or structured [10], and can include senior faculty as guest speakers or facilitators [11].

Group peer mentorship is well suited to improve the transition to attending physician. Peer mentorship groups can address the unique learning needs of new attendings such as research skills, conflict resolution, and teaching skills [10–12]. Peer groups serve a social function and promote the development of long-lasting relationships, which can reduce isolation and improve morale [10, 11, 13]. Peer support is a common function of these groups, where members share professional and personal challenges and discuss mutual experiences [12, 13]. The absence of hierarchy in a peer mentorship group may promote more open discourse when compared with didactic mentoring [13]. Peer mentorship groups promote self-reflection which contributes to professional development [10]. Peer mentorship meetings may be easier to schedule and demand less time than traditional mentorship [12].

There is a paucity of data on the implementation of peer mentorship during the transition to attending physician. Most of the studies to date have involved structured peer mentorship groups which rely on senior faculty facilitators [12, 13]. One study of a peer mentorship group with new psychiatry attendings used an informal format where the agenda was set by the group’s members, although the programme was initiated by a senior faculty member who attended the meetings as an observer [10]. To the best of our knowledge, there is no literature describing a peer mentorship group initiated and lead by new attendings.

When three of us (TM, SR, and JL) graduated and began practising as attendings, we discovered a number of common clinical and non-clinical learning needs. Acting on the advice of a senior faculty mentor (PC), we started a journal club for recent general internal medicine fellowship graduates to address some of these needs. The purpose of the journal club was twofold: first, to facilitate continuing medical education by reviewing the medical literature; second, to provide a forum for peer mentorship by creating a safe space to share fears, discuss challenges, and bond over the joys and frustrations of starting independent practice.

To meet these objectives, we asked members to bring two things to each meeting: a practice-changing journal article, and a ‘transition to practice’ discussion topic such as a diagnostic dilemma, billing question, or a teaching challenge. The most successful venue was a private room at a local pub, where we sat around a long wooden dining table and enjoyed food and drink. Although we briefly considered inviting senior faculty for their advice and mentorship, we decided to limit membership to new attendings to facilitate frank and open discussion.

Beginning in December 2013, we hosted 10 journal club sessions over 14 months. All of the 14 graduates from our fellowship programme in general internal medicine in 2013 were invited to participate. An invitation was subsequently extended to 10 new general internal medicine graduates in 2014 and 6 new attendings who joined our institution from elsewhere. The journal club’s composition varied over time, and members participated in as few as 1 session and as many as 10. Approximately 5–10 individuals attended each session.

We intentionally began each session with unstructured time of 30–60 min for socializing and informal discussion. Each member then presented his or her ‘practice-changing’ article and ‘transition-to-practice’ issue. There was additional time provided at the end of the session for discussion. Our meetings stretched well beyond the allotted two hours and often late into the evening, which we suspect reflects the unmet need for peer mentorship in our group. While confidentiality was not a specific precondition of our sessions, group members indicated in advance if they were discussing a topic that they preferred remain confidential. Discussion of specific patient cases was anonymous with identifying information omitted.

We quickly observed that ‘transition to practice’ issues dominated over journal articles as topics of discussion. This likely occurred because new attendings feel more confident in their clinical knowledge than in other areas such as negotiation, conflict resolution, research, and teaching, which were the focus of the ‘transition to practice’ discussions [2–4, 6].

Conceptually, the discussions on transition to practice fit into three distinct categories that we have termed: trading war stories, measuring up, and navigating uncharted waters (Table 1). A war story is ‘a story of a personal experience that usually involves danger, struggle, or adventure’ [14]. Often, discussions of this nature involved a challenging diagnosis or an unexpected patient outcome. The storyteller frequently framed the experience in terms of a battle, whether with an elusive diagnosis, a disagreement with another physician, or a conflict with a patient or family member. A certain pride was exhibited in establishing a rare diagnosis or working through a challenging administrative or political situation.

**Table 1:** Themes of discussions in a group of physicians transitioning from residency into independent practice

**Theme**	**Specific topics or examples**
War stories	i. Diagnostic challenge or rare diagnosis ii. Unexpected or adverse patient outcome iii. Ethical dilemma iv. Communication challenge (colleague or patient) v. Regulatory college or legal complaint
Measuring up	i. Clinical work (location, time, setting) ii. Practice management (billing, administrative support) iii. Continuing medical education (resources, conferences) iv. Academic work (teaching, research, publications) v. Career management (job negotiations, curriculum vitae) vi. Mentorship (number, quality, functions)
Navigating uncharted waters	i. Transferring a patient from another hospital ii. Giving a negative evaluation to a student iii. Writing a reference letter

Through sharing these war stories, we taught each other about the art as well as the science of medicine. Some stories warned of potential pitfalls, while others solicited feedback, support, or reassurance. A few revealed potentially traumatic experiences, such as adverse patient outcomes, complaints to regulatory agencies, or legal proceedings. Perhaps sharing these stories in a supportive environment had therapeutic benefits for the tellers. Discussions often continued after the journal clubs, either in person, by e‑mail or by text message, and frequently with follow-up discussions at subsequent meetings.

‘Measuring up’ was the second transition to practice theme at the journal club. Early-career physicians need to closely monitor and measure their progress against others. We compared all aspects of our clinical work, practice structure, academic commitments, continuing medical education strategies, and mentorship relationships (Table 1). Discussions frequently revolved around salary, work hours and contract negotiation. The over-riding theme was: ‘How am I doing in relation to my peers?’ These meetings provided a means to periodically evaluate one’s progress and benchmark against colleagues.

The third theme was ‘navigating uncharted waters.’ New situations, or ‘disruptive novel elements,’ often arise unexpectedly in the early stages of one’s medical career [2]. Examples include writing a reference letter, giving a negative evaluation to a student, or responding to an adverse event where a patient experienced harm. Frequently, these situations were not encountered during residency and were not covered in the medical curriculum. We taught each other strategies for navigating these uncharted waters, and mentally rehearsed how we might approach similar situations should they arise in our own practices.

The information shared at our meetings revealed a ‘hidden curriculum’ of learning to become an effective independent practitioner. The information we exchanged could be of local nature, such as which consultants to seek out or avoid at a particular hospital, or it could be of broader applicability, such as the optimal communication strategy to employ when phoning a surgical colleague at 3 A.M. to discuss a contentious case.

In recent months, attendance at the journal club has waned slightly. Competing demands from work and personal lives may be a factor, or it may be that the need for intensive moral support has eased with our growing clinical confidence and evolving career trajectories. With increasing distance from our medical training, we are beginning to recognize common medical knowledge gaps and are considering focusing our meetings more specifically on continuing medical education. Inviting experienced clinicians to future meetings may be one way to accomplish this. We also wonder whether our programme could be expanded to the next generation of new attendings at the University of Toronto who might find this sort of programme useful. Nevertheless, the collegial peer relationships that developed in the group remain robust, and are perhaps one of the most important revenues from our journal club.

We have learned that physicians have a keen need for peer mentorship in the first few years after the transition from residency. An informal discussion group such as a journal club may be one way to fulfil this need. By providing an invaluable forum for ‘trading war stories’, ‘measuring up’, and helping each other ‘navigate uncharted waters,’ our journal club facilitated peer mentorship. We are hopeful that new graduates both at the University of Toronto and elsewhere might consider picking up the baton to keep such programmes fresh and innovative even as we move on to more senior roles.
